# Executive Functions in Overweight and Obese Treatment-Seeking Patients: Cross-Sectional Data and Longitudinal Perspectives

**DOI:** 10.3390/brainsci12060777

**Published:** 2022-06-14

**Authors:** Marco La Marra, Ines Villano, Ciro Rosario Ilardi, Mario Carosella, Maria Staiano, Alessandro Iavarone, Sergio Chieffi, Giovanni Messina, Rita Polito, Chiara Porro, Alessia Scarinci, Vincenzo Monda, Marco Carotenuto, Girolamo Di Maio, Antonietta Messina

**Affiliations:** 1 Department of Experimental Medicine, University of Campania “Luigi Vanvitelli”, 80138 Naples, Italy; ines.villano@unicampania.it (I.V.); cirorosario.ilardi@unicampania.it (C.R.I.); mariocarosella93@gmail.com (M.C.); staianomaria@yahoo.it (M.S.); sergio.chieffi@unicampania.it (S.C.); vincenzo.monda@unicampania.it (V.M.); girolamo.dimaio@unicampania.it (G.D.M.); antonietta.messina@unicampania.it (A.M.); 2 Department of Psychology, University of Campania “Luigi Vanvitelli”, 81100 Caserta, Italy; 3 Neurological Unit, CTO Hospital, AORN “Ospedali dei Colli”, 80131 Naples, Italy; aleiavarone@gmail.com; 4 Department of Clinical and Experimental Medicine, University of Foggia, 71122 Foggia, Italy; giovanni.messina@unifg.it (G.M.); rita.polito@unifg.it (R.P.); chiara.porro@unifg.it (C.P.); 5 Department of Education Sciences, Psychology, and Communication, University of Bari, 70121 Bari, Italy; alessia.scarinci@unifg.it; 6 Clinic of Child and Adolescent Neuropsychiatry, Department of Mental Health, Physical and Preventive Medicine, University of Campania “Luigi Vanvitelli”, 80138 Naples, Italy; marco.carotenuto@unicampania.it

**Keywords:** obesity, executive functions, weight loss, inhibition, verbal fluency, psychomotor speed

## Abstract

Background: Recent evidence suggests that a higher body weight may be linked to cognitive impairment in different domains involving executive/frontal functioning. However, challenging results are also available. Accordingly, our study was designed to verify whether (i) poor executive functions are related to a higher body weight and (ii) executive functioning could contribute to weight loss in treatment-seeking overweight and obese patients. Methods: We examined general executive functioning, inhibitory control, verbal fluency, and psychomotor speed in a sample including 104 overweight and obese patients. Forty-eight normal-weight subjects participated in the study as controls. Results: Univariate Analysis of Variance showed that obese patients obtained lower scores than overweight and normal-weight subjects in all executive measures, except for errors in the Stroop test. However, when sociodemographic variables entered the model as covariates, no between-group difference was detected. Furthermore, an adjusted multiple linear regression model highlighted no relationship between weight loss and executive scores at baseline. Conclusions: Our results provide further evidence for the lack of association between obesity and the executive domains investigated. Conflicting findings from previous literature may likely be due to the unchecked confounding effects exerted by sociodemographic variables and inclusion/exclusion criteria.

## 1. Introduction

Obesity is a chronic illness that can lead to an increased risk of lower quality of life and premature death [1]. Body fat, besides representing a major risk factor for different chronic diseases such as type II diabetes mellitus, fatty liver disease, hypertension, myocardial infarction, stroke, osteoarthritis, kidney disease, dyslipidemias, obstructive sleep apnea and cancer [2,3], may also represent a significant predictor of impaired cognitive performance, accelerated cognitive decline, and dementia [4,5,6,7,8,9,10,11,12,13,14]. In this vein, many studies have explored the possible link binding executive functions to body weight. Executive functions (EFs) are generally considered as higher cognitive processes that enable forethought and goal-directed actions [15]. They involve different domains (e.g., attention, processing speed, set-shifting, inhibitory control, working memory, concept formation, problem solving) [15,16,17] primarily mediated by neural activity in the prefrontal cortex (PFC) [18,19]. These domains support goal-directed behaviors [20] and are recruited in situations requiring adaptation to cope with environmental demands in unfamiliar or conflicting contexts [15,16]. Particularly, EFs allow inhibition of strong dominant responses/interfering stimuli, or resistance to temptations [20].

Differences in executive performance may affect lifestyle habits and predispose individuals to excessive body weight [21]. Lower EFs were found to be significantly associated with some dysfunctional eating-related behaviors, e.g., weak food inhibition [22,23,24], greater intake of fatty foods [25], reduced control in appetite regulation [26], less physical activity [27], higher emotional eating [28], inability to learn from errors or past experiences [29], delay in weight loss [22], poorer adherence to dietary intentions [30], and poor treatment outcomes during weight loss interventions [31]. In recent decades, some studies have shown that obesity was related to impaired performance on tasks assessing EFs [21,32,33]. It has been argued that obese subjects would be unable to delay gratifications or inhibit prepotent responses to highly palatable foods [33]. However, other studies were inclined to sustain the opposite point of view, with obese subjects showing equal or better executive performance than normal-weight subjects [34,35,36,37]. These findings would support the rationale of the so-called “obesity paradox”, i.e., excess adiposity as a protective factor for health outcomes including cognitive functioning and mortality, especially in geriatric patients [37,38,39]. Although previous research on the relationship between obesity and EFs has provided conflicting evidence on most of the executive domains explored [21,26,32,33,40,41], many concerns remain about inhibitory control, verbal fluency, and psychomotor speed domains, especially when the potential moderation effect of covariates has not been taken into account.

Inhibitory control refers to the ability to voluntarily suppress interfering information or prepotent/habitual responses in line with task demands. Some studies have reported lower inhibitory control in obese than normal-weight subjects [21,22,42]. Conversely, other investigations did not find any relationship between BMI and inhibitor control [32,43,44,45].

Verbal fluency refers to the ability to spontaneously retrieve specific information within phonemic or semantic constraints [46,47]. Some studies exploring verbal fluency abilities found that obese subjects performed worse than non-obese [35,48]. On the contrary, other studies showed no between-group difference [49] or reported higher scores in obese participants when compared with normative data [21,32,33,34].

Finally, psychomotor speed refers to the ability to detect, and respond to, rapid changes in the environment, such as the presence of a stimulus [47,50]. Some studies have found significantly poorer performance in obese subjects compared with controls [51], whereas others have failed to find difference in psychomotor speed between obese and normal-weight subjects [52]; still others have shown that obese participants obtained better performance than normal-weight subjects [37].

In addition, adherence to weight loss programs and the maintenance of the achieved body weight over time is determined by the individual’s ability to self-manage [53]. It is characterized by realistic goal setting, adequate self-control, problem-solving skills, and proper planning of actions to be taken [54,55]. These skills represent higher-order cognitive processes belonging to EFs. Indeed, it has been suggested that impairment of EFs might be related to difficulties in adhering to dietary prescriptions [33]. However, to the best of our knowledge, weak evidence is available on the matter.

In order to disentangle the relationship between obesity and EFs, the present study was designed to verify whether (i) a difference exists on EFs between normal-weight, overweight and obese subjects computing the potential effects exerted by sociodemographic characteristics (i.e., sex, age, education) and whether (ii) executive performance could predict weight change—in terms of BMI percentage—following dietary prescription in overweight and obese treatment-seeking patients. We hypothesize statistically significant differences in the executive domains investigated. Furthermore, we expect that these domains are able to predict weight loss.

## 2. Materials and Methods

### 2.1. A Priori Power Analysis

G*Power 3.1.9.4 was used to perform an a priori power analysis for determining the number of participants needed according to our multidimensional statistical approach based on a generalized linear model. As for analysis of variance (ANOVA), at a nominal alpha level (α) of 0.05, power (1 − β) set to 0.80, large effect size (*f* = 0.40), and number of groups set to 2 or 3, the required total sample size (*N*_r_) was estimated to be between 52 and 66. As for analysis of covariance (ANCOVA), at a nominal α of 0.05, 1 − β set to 0.80, *f* = 0.40, and both number of groups and covariates set to 3 (*df* = 2), the computed *N*_r_ was 64. Finally, multiple linear regression analysis was set at a nominal α of 0.05, 1 − β of 0.80, large effect size (*f*^2^ = 0.35), and 5 predictors, *N*_r_ 43.

### 2.2. Participants

One hundred and four treatment-seeking overweight and obese patients (58 females, *M* age = 36.40, *SD* = 12.41; *M* education = 10.83, *SD* = 3.19; *M* BMI = 31.36, *SD* = 3.89) took part in this study as participants. They were recruited at the Department of Experimental Medicine (Section of Human Physiology and Human Dietetic Service) of the University of Campania “Luigi Vanvitelli”. The inclusion/exclusion criteria were: absence of intellectual or linguistic deficits, absence of neurological, psychiatric, or psychopathological disorders (e.g., schizophrenia, TIA, stroke, head trauma, epilepsy, major depressive disorder, bipolar disorder), non-progressive (e.g., post-traumatic) or reversible (e.g., metabolic-type, by substance intoxication, by nutritional deficiencies) dementia, and no history of alcohol or drug abuse/addiction. Furthermore, no participant satisfied diagnostic criteria for metabolic syndrome or eating disorders. To mitigate the effects of potential comorbidities, subjects with BMI > 40 were excluded. In accordance with the inclusion and exclusion criteria, 48 normal-weight subjects were included as control participants.

The anthropometric measurements (i.e., weight and height) of each participant were detected. According to Quetelet’s formula (kg/m^2^), three subgroups were ranked based on BMI (normal-weight, overweight, and obese). The whole sample included 48 normal-weight (26 females, *M* BMI = 23.73, *SD* = 1.56; *M* age = 30.00, *SD* = 6.79; *M* years of education = 12.58, *SD* = 1.39), 26 overweight (10 females, *M* BMI = 27.25, *SD* = 1.11; *M* age = 34.38, *SD* = 10.68; *M* years of education = 13.00, *SD* = 0.30), and 78 obese subjects without eating disorders (48 females, *M* BMI = 34.17, *SD* = 2.21; *M* age = 37.08, *SD* = 12.93; *M* years of education = 10.10, *SD* = 3.39).

### 2.3. Procedure

To fulfill our first aim, sociodemographic (i.e., sex, age, and years of education) and anthropometric data (i.e., BMI) were collected for each patient. Contextually, patients were administered a brief neuropsychological battery to assess general and specific executive functioning. Patients’ performance on executive tasks was compared with that of a control group consisting of normal-weight subjects. To fulfill our second aim, we compared patients’ BMI, detected at baseline, with that calculated at six-months follow-up after a tailored diet plan, based on the principles of the hypocaloric Mediterranean diet.

### 2.4. Measures

The neuropsychological assessment of EFs included a measure of general functioning, i.e., the Frontal Assessment Battery–15, and more targeted measures exploring subdomains including impulsivity/inhibitory control (i.e., Stroop Color-Word Test), cognitive flexibility (i.e., FAS verbal fluency test), and psychomotor speed (i.e., Digit Symbol Substitution Test).

*Frontal Assessment Battery–15* (FAB15) [19]. The FAB15 is a short neuropsychological screening battery providing a quick, valid and reliable estimate of general executive functioning. The FAB15 demonstrated good internal consistency (Cronbach’s α = 0.72), solid factorial structure, and excellent interrater (ICC = 0.99) and test–retest reliabilities (ICC = 0.98).

*Stroop Color-Word Test* (SCWT) [56]. This is the most widely used cognitive task to assess the ability to inhibit interference from a dominant response tendency. Previous literature has reported its application for the assessment of other cognitive domains such as attention, processing speed, cognitive flexibility, and working memory [57,58]. The total number of errors and completion time for the entire task served as dependent variables.

*FAS Verbal Fluency Test* (FAS) [47,59,60]. The FAS test is a measure of phonemic fluency requesting the participant to produce as many words as possible that begin with letters “F”, “A” and “S” within one minute each. Successive retrieval requires executive control over cognitive processes, e.g., selective attention, set-shifting, generativity, and self-monitoring. The total number of correct words represent the dependent variable.

*Digit Symbol Substitution Test* (DSST) [61,62]. This is a pencil-and-paper test commonly employed to assesses psychomotor speed, although it taps into other cognitive processes such as processing speed, set-shifting, working memory, associative and implicit learning. Furthermore, the DSST may also be used as a sensitive measure of decision making in clinical settings [62]. The participant is presented with a grid of numbers and matching symbols under which there is a test section with numbers and empty boxes. The test consists of filling as many empty boxes as possible with the appropriate symbol. The number of correct number-symbol matches completed in 90 s is scored.

## 3. Results

Univariate outliers (i.e., z-scores = |3|) were removed. If needed, square root transformation (√X_i_) was performed to normalize variables in line with skewness and kurtosis parameters (i.e., <|1|). For multivariate diagnostics of outliers, the Mahalanobis distance ($D_{i}^{2}$) was calculated. Accordingly, no multivariate outliers were detected ($D_{i}^{2}$ = 16.57, df = 8, *p* < 0.001). Multivariate normality was assumed by Mardia’s coefficient $\left(\;\frac{\sum_{i=1}^{N}{\left(D_{i}^{2}\right)}^{2}\;}{N}\;\right)$ = 74.99 < 80. Missing data were analyzed and random missingness (MCAR) was detected. Therefore, we adopted the recommended multiple imputation in order to treat missing data.

### 3.1. Descriptive Statistics

Sample characteristics are summarized in Table 1. No difference in the frequency of gender levels was detected in the three BMI subgroups (χ^2^_(2)_ = 4.235, *p* = NS, φ = 0.18). Results of Univariate ANOVA highlighted a significant effect of sex on BMI (F_(1, 150)_ = 5.912, *p* = 0.02, η^2^ = 0.04), with female participants showing higher BMI score (female, M BMI = 34.49, SD = 10.25 vs. male, M BMI = 30.83, SD = 7.82). No gender difference was found in any executive score (FAB15, F_(1, 150)_ = 0.071, *p* = NS), FAS (F_(1, 150)_ = 2.712, *p* = NS), DSST (F_(1, 150)_ = 1.597, *p* = NS), Stroop-T (F_(1, 150)_ = 0.497, *p* = NS), and Stroop-E (F_(1, 150)_ = 3.805, *p* = NS). A significant effect of age was found on BMI according to Univariate ANOVA (F_(2, 149)_ = 6.195, *p* = 0.03, η^2^ = 0.07). Bonferroni’s post-hoc analysis showed that obese subjects were older than normal-weight subjects (mean difference = 7.077, SE = 2.01, *p* = 0.02). Similarly, as for years of schooling, a between-group difference emerged (F_(2, 149)_ = 20.255, *p* < 0.001, η^2^ = 0.21), with obese subjects being less educated than normal-weight (mean difference = –2.481, SE = 0.47, *p* < 0.001) and overweight subjects (mean difference = –2.897, SE = 0.58, *p* < 0.001). About the effects of age and education on executive scores, our results confirmed their principal effects, with higher age and lower education affecting all scores (all p_s_ < 0.0001). Results of Univariate ANOVAs showed that obese subjects reported worst scores on FAB15 (F_(2, 149)_ = 5.834, *p* < 0.01), FAS (F_(2, 149)_ = 9.033, *p* < 0.001), DSST (F_(2, 149)_ = 14.818, *p* < 0.001), and Stroop-T (F_(2, 149)_ = 7.769, *p* = 0.01). Conversely, no between-group difference in Stroop-E (F_(2, 149)_ = 2.495, *p* = NS) was detected. Raw scores obtained on each neuropsychological test are reported in Table 1. Given the concomitant effects of sex, age and education, successive analyses will be adjusted accordingly.

### 3.2. Multivariate Analysis of Covariance in Baseline Measurements

Results of MANCOVA showed no significant effect of BMI group (F_(10, 284)_ = 0.976, *p* = NS) on executive scores after adjusting for the significant contribution of sociodemographic variables (sex, F_(5, 142)_ = 4.274, *p* < 0.01; age, F_(5, 142)_ = 14.886, *p* < 0.001; education, F_(5, 142)_ = 8.310, *p* < 0.001). As a consequence, Univariate ANCOVAs revealed no difference between groups on FAB15 (F_(2, 146)_ = 0.02, *p* = NS), FAS (F_(2, 146)_ = 0.723, *p* = NS), DSST (F_(2, 146)_ = 1.231, *p* = NS), Stroop-T (F_(2, 146)_ = 1.651, *p* = NS), and Stroop-E (F_(2, 146)_ = 0.664, *p* = NS). Results of each ANCOVA are reported in Table 2.

### 3.3. Multiple Linear Regression Analysis and Relationships to Weight Loss

Upon follow-up period of six months, overweight and obese participants demonstrated, on average, significant weight reduction in terms of BMI (–5.46%, *p* < 0.001), suggesting good adherence to diet therapy. In order to investigate whether baseline executive scores predicted the decreasing weight, we ran a multiple linear regression analysis where the BMI change (i.e., the difference between the BMI at follow up and BMI at baseline) entered the model as dependent variable, whereas the five raw executive scores entered as predictors. Each executive score was entered in the model after undergoing adjustment according to sex, age, and education by typesetting the following correction equation:

$$Raw\;score-\left[B_{sex}\times \left(x_{i\left(sex\right)}-{\bar{x}}_{\left(sex\right)}\right)\right]-\left[B_{age}\times \left(x_{i\left(age\right)}-{\bar{x}}_{\left(age\right)}\right)\right]-\left[B_{edu}\times \left(x_{i\left(edu\right)}-{\bar{x}}_{\left(edu\right)}\right)\right]$$

As shown in Table 3, the regression model was not significant (F_(5, 98)_ = 3.217, *p* = NS), with none of the scores being able to explain a sufficient portion of the variance in the BMI change (FAB15, *B* = 0.002, *t* = 0.018, *p* = NS; FAS, *B* = −0.002, *t* = –0.152, *p* = NS; DSST, *B* = −0.012, *t* = −0.886, *p* = NS; Stroop-T, *B* = 0.062, *t* = 1.892, *p* = NS; Stroop-E, *B* = 0.244, *t* = 0.713, *p* = NS).

## 4. Discussion

In the current study, we compared performance of obese, overweight, and normal-weight participants on neuropsychological tasks exploring general and specific (i.e., inhibition/impulsive control, verbal fluency, and psychomotor speed) executive functioning.

In a scientific context of conflicting evidence, our findings showed that obese individuals did not report poorer executive performance when compared to over- and normal-weight subjects in a general linear model adjusted for sex, age, and education levels. We found also that covered executive scores did not predict weight loss in treatment-seeking overweight and obese individuals.

Different interacting factors may explain the null results we found: the complexity and amplitude of the neural circuits underlying EFs, the role of comorbidities likely to be mediating the obesity vs. EFs relationship, and the removal of the effects exerted by demographic variables.

Regarding the complexity and extent of neural circuits, it should be noted that the integrity of the executive domains explored in the present study appears to depend on the activity across a wide brain network, mainly including frontal regions. For instance, inhibitory control involves dorsolateral, ventromedial, orbital prefrontal and anterior cingulate cortices [63,64]. Performance in verbal fluency tasks has been found to correlate with activity in medial frontal areas, inferior frontal gyrus, anterior and posterior cingulate cortices [65,66]. Finally, psychomotor speed seems to rely on the frontoparietal network, particularly involving the middle frontal gyrus and the posterior parietal cortex [67,68,69]. However, functional connectivity within the PFC, as well as between PFC and some subcortical regions (e.g., basal ganglia, subthalamic nucleus, hippocampal formation), may also play a crucial role, particularly in patients with eating disorders and/or severe obesity [63,64,65,66,70,71]. Although these circuits deserve to be explored in clinical populations (e.g., brain-injured or psychopathological patients), neuropsychological tasks—which have been traditionally devised to detect cognitive impairments—may show low sensitivity and specificity in non-clinical subjects. 

In individuals showing aberrant eating behaviors, projections between PFC and hypothalamic circuits appear relevant to modulation of hunger and satiety signals, while striatal and ventral midbrain circuits seem to be relevant for reward processing [64,72,73]. Moreover, in these patients, such neural circuits could affect feeding behaviors, leading to difficulty in planning regular eating patterns, and inability to delay gratification or inhibit prepotent responses to highly palatable foods [33]. Still, there is evidence of pronounced impairment of EFs in adults with more complicated obesity profiles (e.g., metabolic syndrome or clinical experiences of loss of control eating) [74]. For instance, it has been hypothesized that deficits in some executive domains (e.g., cognitive inflexibility, self-regulation, planning, inhibition) are likely to lead to binge eating episodes, a potential behavioral risk factor for severe obesity [75,76,77]. The capacity to switch among cognitive strategies in response to changing environments or rules also involves the ability to overcome or suppress previously learned thinking/behavior patterns to adaptively deal with new situations. Accordingly, patients with eating disorders frequently present with inflexible thought and rigid behavioral patterns, particularly (but not exclusively) surrounding food and feeding behaviors [64].

It is important to underline that some investigations did not report relevant information characterizing the study sample, such as clinical status of participants, education, intake of any obesity-treating medication, or presence of psychiatric disorders [21]. This lack of transparency and methodological rigor makes it unclear whether obesity actually predisposes to cognitive impairments since some covariates (socioeconomic status, vascular disease, genetics) may exercise a confounding/mediating action. For instance, obesity increases the risk of hypertension, diabetes [78], stroke and leptin dysregulation [79,80] that may affect, per se, cognitive performance. Interestingly, recent research has proposed that BMI and EFs may be indirectly associated via obesity-induced activation of innate immunity as a result of a low-grade inflammation process (e.g., abnormal adipokine and cytokine secretion such as TNF-alpha and interferon [81,82,83,84,85,86,87], as furthered by a newly proposed model, the immunologic model of self-regulatory failure [81,83,88].

Additional confounds contaminating the relationship between obesity and EFs are socio-demographic variables, especially since neuropsychological tasks are extremely sensitive to these parameters. Cognitive changes as a result of normal aging has been well-documented in the scientific literature. Some abilities are resistant to brain aging, whereas others (e.g., memory, EFs, visuospatial abilities, language) decline gradually over time [89]. Low education level has also been shown as an independent risk factor for cognitive impairment, whereas high education level plays a protective role [90,91,92]. Finally, gender differences in cognitive performance have often been reported. As for EFs, the interaction between sex, brain circuits, education, and occupational levels may explain the superiority of men in some executive tasks, e.g., planning and inhibition, and why women instead outperform men in tests exploring other executive domains such as verbal fluency (see [19] for an overview of the matter).

Finally, in line with recent research [53], we found no relationship between weight loss and executive scores collected at baseline. However, this result may reflect our patients’ characteristics. Indeed, our sample consisted of subjects who voluntarily required dietary intervention, and therefore our results are not generalizable to not-treatment seeking patients. Interestingly, it has been suggested [93] that intentional weight loss might be related to increased cognitive abilities in several domains; therefore, adherence to a restrictive diet might predict an improvement in executive functioning.

Our study presents some limitations, particularly in terms of external validity. First, we focused only on the sociodemographic variables (i.e., sex, age, and education) that are notoriously considered significant confounding factors for neuropsychological scores. Nevertheless, other relevant confounds should be accounted for as potential covariates (e.g., race, household income, marital status). Second, patients with BMI > 40 were excluded from the analyses as they did not meet our inclusion criteria (e.g., they suffered from metabolic syndrome or neurological/psychiatric diseases); furthermore, morbid obesity is often related to several comorbidities potentially affecting cognitive performance. However, further studies could assess EFs in this specific population. Third, another limitation is the adoption of BMI for discriminating of subgroups. Indeed, other indicators for obesity, such as waist-to-hip ratio, could be more reliable in predicting executive performance [94]. Finally, future studies including a larger number of participants are needed to increase statistical power and thus the detection of potential small effect sizes.

## 5. Conclusions

Our results provide further evidence for the lack of association between obesity and EFs. Conflicting findings from previous literature may likely be due to the unchecked confounding effects exerted by sociodemographic variables and inclusion/exclusion criteria. Future studies should focus on morbid obesity and extend the follow-up period.

## Figures and Tables

**Table 1:** Sample characteristics and raw performance on executive tasks for each group.

	Normal-Weight (*n* = 48)	Overweight (*n* = 26)	Obese (*n* = 78)	Sig.
BMI, mean (SD)	23.73 (1.56)	27.25 (1.11)	37.16 (3.31)	
Sex (f/m)	26/22	10/16	48/30	
Age, mean (SD)	30.00 (6.79)	34.38 (10.68)	37.08 (12.93)	*
Education, mean (SD)	12.58 (1.39)	13.00 (0.30)	10.10 (3.39)	***
FAB15, mean (SD)	13.00 (1.77)	13.50 (1.39)	11.83 (2.43)	**
FAS, mean (SD)	45.35 (6.97)	44.75 (9.89)	36.65 (12.16)	***
DSST, mean (SD)	59.04 (12.09)	61.15 (11.52)	46.57 (17.46)	***
Stroop-T, mean (SD)	13.42 (8.21)	12.17 (6.82)	18.92 (10.67)	**
Stroop-E, mean (SD)	0.36 (0.63)	0.54 (0.61)	0.62 (0.62)	

BMI: Body Mass Index; FAB15: Frontal Assessment Battery–15; FAS: FAS Verbal Fluency Test; DSST: Digit Symbol Substitution Test; Stroop-T: Stroop Color-Word Test-Time; Stroop-E: Stroop Color-Word Test-Error. Note: * *p* < 0.05, ** *p* < 0.01, *** *p* < 0.001. Age and Education are expressed in years.

**Table 2:** Results of Univariate Analyses of Covariance.

	Sum of Square	*F*	Sig.	*η^2^*
BMI * FAB15	0.09	0.02		0.001
Sex	1.25	0.54		0.007
Age	1.68	0.73		0.010
Education	41.26	17.77	***	0.194
BMI * FAS	103.72	0.72		0.019
Sex	61.16	0.85		0.011
Age	2.57	0.04		0.001
Education	1194.51	16.65	***	0.184
BMI * DSST	252.95	1.23		0.032
Sex	37.32	0.36		0.005
Age	4761.68	46.35	***	0.385
Education	2995.76	29.16	***	0.283
BMI * Stroop-T	0.89	1.65		0.043
Sex	3.21	11.902	**	0.139
Age	4.83	17.94	***	0.195
Education	1.15	4.27	*	0.055
BMI * Stroop-E	43.64	0.66		0.018
Sex	33.73	0.03		0.014
Age	1343.18	40.87	***	0.356
Education	267.82	8.15	**	0.099

BMI: Body Mass Index; FAB15: Frontal Assessment Battery–15; FAS: FAS Verbal Fluency Test; DSST: Digit Symbol Substitution Test; Stroop-T: Stroop Color-Word Test-Time; Stroop-E: Stroop Color-Word Test-Error. Note: * *p* < 0.05, ** *p* < 0.01, *** *p* < 0.001.

**Table 3:** Results of Multiple Linear Regression Analysis on BMI change.

Predictors	*B*	95% CI		*SE*	*t*	*p*
		LL	UL			
FAB15	0.002	–0.218	0.222	0.11	0.018	0.99
FAS	–0.002	–0.034	0.029	0.02	–0.152	0.88
DSST	–0.012	–0.040	0.016	0.01	–0.886	0.38
Stroop-T	0.062	–0.004	0.128	0.03	1.892	0.11
Stroop-E	0.244	–0.446	0.933	0.34	0.713	0.48

FAB15: Frontal Assessment Battery–15; FAS: FAS Verbal Fluency Test; DSST: Digit Symbol Substitution Test; Stroop-T: Stroop Color-Word Test-Time; Stroop-E: Stroop Color-Word Test-Error. Note: Predictors entered the regression model after normative adjustment for sex, age, and education according to the following correction formula; $Raw\;score-\left[B_{sex}\times \left(x_{i\left(sex\right)}-{\bar{x}}_{\left(sex\right)}\right)\right]-\left[B_{age}\times \left(x_{i\left(age\right)}-{\bar{x}}_{\left(age\right)}\right)\right]-\left[B_{edu}\times \left(x_{i\left(edu\right)}-{\bar{x}}_{\left(edu\right)}\right)\right]$.

## Data Availability

The data presented in this study are available on request from the corresponding author.
